# Deciphering the association between mortality salience and the “lying flat” movement: a social media analysis

**DOI:** 10.3389/fpsyg.2026.1854408

**Published:** 2026-08-07

**Authors:** Jingyuan Zhu, Kaiyang Qin, Fangyuan Liu, Yue Hu, Hua Pang

**Affiliations:** 1 Counseling and Education Center, Xiamen University, Xiamen, China; 2 School of Law and Economics, Wuhan University of Science and Technology, Wuhan, China; 3 Key Laboratory of Adolescent Cyberpsychology and Behavior (CCNU), Ministry of Education, Wuhan, China; 4 Key Laboratory of Human Development and Mental Health of Hubei Province, School of Psychology, Central China Normal University, Wuhan, China; 5 The Nethersole School of Nursing, The Chinese University of Hong Kong, Hong Kong, Hong Kong SAR, China; 6 School of New Media and Communication, Tianjin University, Tianjing, China

**Keywords:** adaptive topic, defensive topic, lying flat, mortality salience, social media

## Abstract

**Introduction:**

When individuals face overwhelming situations, such as the heightened awareness of mortality triggered by the pandemic, they may adopt the “lying flat” response. “Lying flat,” a movement on Chinese social media platforms, refers to the adoption of a minimalist lifestyle and the rejection of intensive life or work goals. However, the psychological mechanisms driving this phenomenon remain underexplored. Drawing on psychological perspectives of existential threat and coping strategies, this study aims to investigate whether mortality salience is associated with “lying flat” discourse and to determine if it functions primarily as a defensive or adaptive coping strategy.

**Methods:**

We analyzed 257,410 Weibo posts to examine the association between mortality salience and “lying flat” discourse on Weibo. Mortality salience was operationalized using pandemic death counts. We employed a keyword-assisted topic modeling approach to categorize the Weibo posts into defensive versus adaptive coping strategies. Crucially, we utilized stratified regression analyses to examine the association between mortality salience and the proportions of defensive and adaptive topics.

**Results:**

The results revealed that mortality salience was positively associated with the overall frequency of “lying flat” engagement, and users predominantly engaged in defensive coping mechanisms. Furthermore, stratified regression demonstrated that higher death tolls were significantly associated with an increased proportion of defensive topics (e.g., immediate gratification and avoidance) but a decrease in adaptive topics (e.g., re-evaluating life goals).

**Discussion:**

These findings suggest that digital “lying flat” may serve as a psychological buffer to mortality-related stress. Our results may indicate an asynchrony in these mechanisms, where adaptive responses emerge more slowly than immediate defensive reactions. We highlight the distinction between merely expressing “lying flat” and actual behavioral withdrawal. Finally, we underscore the importance of future cross-cultural research to comprehensively understand the global manifestations of such phenomenon under existential crises.

## 1 Introduction

Mortality salience, as defined by Greenberg et al. (1990), involves the acknowledgment and comprehension of one’s mortality. Individuals who perceive mortality salience may respond in an avoidant or defensive manner (Greenberg et al., 1994), including shortsighted behavior for immediate rewards and high-risk behavior (e.g., excessive consumption or risky driving) (Hirschberger et al., 2002; Lerner et al., 2012; Rosenbloom, 2003). In some contexts, mortality salience can also catalyze adaptive behaviors, such as exploration of alternative beliefs and values (e.g., pondering the meaning of life) (Greenberg et al., 2008). As a global crisis resulting in hundreds of thousands of deaths, the COVID-19 pandemic has become a significant source of triggering mortality salience (Pyszczynski et al., 2020). One potential outcome of such widespread mortality salience is likely a reduction in work intensity and engagement (e.g., Liu et al., 2021). These reactions, coupled with manifestations such as diminished desires and a tempered pursuit of ambitions, have been collectively termed “lying flat” on Chinese social media platforms (Wang and Yao, 2023). The present study aims to investigate the association between COVID-19-induced mortality salience and the prevalence of the “lying flat” movement on Chinese social media.

“Lying flat” represents a lifestyle choice characterized by resisting societal pressures to pursue success, focusing on minimizing or eschewing professional challenges and ambitious goals (Hsu, 2022). Previous studies have indicated that the “lying flat” movement is closely associated with social-economic factors, including unemployment rates (Wu and Sun, 2024), family economic background (Noh and Lee, 2017), and peer pressures (Wang et al., 2024). In a challenging economic climate, individuals may become increasingly concerned about their careers and future. They may feel that, despite their relentless efforts, the status quo remains unchangeable. This mindset often leads to a diminished desire to pursue personal aspirations. In this sense, some researchers suggest that this “lying flat” phenomenon serves as a coping mechanism, a strategic response to mitigate feelings of powerlessness (Wu and Sun, 2024).

More importantly, some researchers perceive “lying flat” as a defensive and pessimistic approach to life. For example, Deng et al. (2022) suggest that “lying flat” reflects the psychological distress and powerlessness experienced by contemporary youth amid the internalization of social pressures, which often lead to a pessimistic outlook on life. In contrast, others adopt an adaptive perspective by viewing “lying flat” as a simpler, more natural way of life. For instance, Song and Bie (2022) argue that the “lying flat” mindset toward work and life reflects a self-adaptation process. Namely, individuals who embrace the “lying flat” mindset may consciously downplay societal pressures but prioritize their fundamental needs, such as personal well-being. This approach, therefore, allows them to navigate society and life with less burden. Taking these examples together, we argue that “lying flat” may likely contain both defensive and adaptive dimensions. In other words, defensive “lying flat” is a passive coping strategy in response to external pressures, such as feelings of helplessness induced by the awareness of mortality, via immediate gratification and avoidance. Adaptive “lying flat” is a proactive approach to adopting a “lying flat” lifestyle. It involves prioritizing work-life balance, intentionally reducing work intensity, and embracing personal growth while confronting societal and life challenges.

Taken together, existing studies suggest that “lying flat” is associated with social and economic factors, and these studies reflect both defensive and adaptive aspects of “lying flat.” While existing literature has largely framed “lying flat” as a sociological response to structural issues, such as intense social competition (e.g., Wang et al., 2024), the underlying psychological mechanisms, particularly how existential threats shape this behavior, remain under-explored. While social-economic indicators explain the structural constraints and external triggers of “lying flat”, mortality salience may offer insights into its underlying psychological processes, because it may address how individuals fundamentally recalibrate their life’s worth when confronted with the fragility of existence. As the awareness of death represents an overwhelming stressor (e.g., Menzies et al., 2019), “lying flat” may function as a psychological strategy to cope with this existential threat. Therefore, it is necessary to explore the relationship between mortality salience and “lying flat” from a psychological perspective.

Research has demonstrated that mortality salience can influence individuals’ behavior across various dimensions (Burke et al., 2010). For instance, studies found that it can promote prosocial behavior (Hirschberger et al., 2008), enhance preference for in-groups (Harmon-Jones et al., 1996), and facilitate the pursuit of goals, such as financial success (Arndt et al., 2004). Interestingly, in these studies, two primary responses associated with mortality salience on behavior have been identified: the defensive and the adaptive responses. The defensive responses may originate from the awareness that mortality triggers existential anxiety (Zaleskiewicz et al., 2013). Individuals may respond defensively to anxiety by seeking immediate rewards (Lerner et al., 2012). For example, triggering mortality salience to consumers may increase their desire for high-status products (e.g., luxury cars) rather than low-status products (e.g., budget cars) (Greenberg et al., 1994). In contrast, in some situations, individuals may adopt an adaptive approach. For example, researchers found that participants primed with death scenarios may trigger stronger gratitude for life, compared to those in the control condition (e.g., Frias et al., 2011). The association between mortality salience and adaptive responses has also been demonstrated in some clinical cases. For example, researchers found that post-traumatic growth, such as a reappraisal of life and priorities, and a new awareness of the body, can occur in individuals with life-threatening physical illnesses (Hefferon et al., 2009).

The finding that mortality salience is associated with both defensive and adaptive responses resonates well with the Dual Existential Systems Theory, which proposes that individuals, in response to mortality salience, may exhibit either defensive or adaptive responses depending on the contextual cues that trigger awareness of death (Cozzolino, 2006). According to this theory, abstract concepts of mortality (such as those elicited by responding to open-ended questions about their thoughts and feelings about death) increase defensiveness in one’s own cultural worldview, meaning that reminders of mortality may increase the need for the protection provided by faith in the cultural worldview. For example, a person with strong religious beliefs may become more convinced of their religious doctrine when confronted with death. Yet specific mortality concepts, such as illness or trauma, increased death-related growth, such as eliciting thoughts related to self, life, and significant others. For example, a person being diagnosed with a life-threatening illness might start to prioritize spending more time with loved ones, re-evaluating their life goals, and making significant changes to live more authentically and meaningfully.

To the best of our knowledge, while no study has directly explored the link between mortality salience and the “lying flat” phenomenon, existing research suggests a potential association. Researchers have proposed that the phenomenon of “lying flat” is intricately linked to the anxiety arising from stress (e.g., Lu et al., 2023), meaning that individuals may use “lying flat” as a coping strategy to address their anxiety, such as diminishing the drive for achievement and embracing the status quo. Importantly, one of the unique characteristics of mortality salience is that it normally evokes existential anxiety pertaining to death (Greenberg et al., 1994). Considering that people may perceive global pandemics, such as COVID-19, as an enormous source of mortality salience (e.g., Pyszczynski et al., 2020), it is plausible that individuals may adopt “lying flat” as an existential buffer to mitigate existential anxiety triggered by the pandemic. In the digital age, social media platforms like Weibo serve as a primary space for the articulation of such collective psychological responses, where existential concerns are translated into public discourse (e.g., Wang et al., 2020). Therefore, we hypothesized that:

*H1*: The mortality salience induced by the COVID-19 pandemic is positively associated with the frequency of “lying flat” responses on social media.

Furthermore, considering that mortality salience can elicit both defensive and adaptive responses, depending on the nature of the death reminder (Cozzolino, 2006), it is plausible that mortality salience may be associated with different dimensions of “lying-flat” responses. More specifically, the COVID-19 pandemic represents a multifaceted existential threat that includes both abstract and concrete dimensions. On one hand, daily reports of death tolls and statistical data serve as abstract reminders, which typically trigger defensive responses such as avoidance. On the other hand, the immediate risk of infection and the witnessing of suffering represent concrete stressors that can catalyze adaptive growth, such as the reappraisal of life priorities and the pursuit of intrinsic well-being. Accordingly, we argue that the “lying flat” movement is a multifaceted psychological phenomenon containing both facets: a defensive withdrawal from external pressures and an adaptive realignment toward personal growth. Hence, we hypothesized that:

*H2*: Mortality salience is positively associated with the proportion of defensive “lying flat” topics.

*H3*: Mortality salience is positively associated with the proportion of adaptive “lying flat” topics.

In this study, we aim to examine the extent to which mortality salience is associated with the prevalence of “lying flat” responses on social media. Specifically, we investigate how mortality salience is associated with the defensive and adaptive dimensions of this movement. Mortality salience was operationalized using the number of deaths caused by COVID-19 as a macro-level indicator. This approach aligns with previous research indicating that the number of deaths caused by COVID-19 can function as environmental cues that activate death-related awareness and psychological responses (Rodriguez et al., 2022). The frequency of Weibo posts mentioning “lying flat” was used as an index for engaging in “lying flat” discourse. We implemented a topic modeling approach to identify distinct defensive and adaptive topics within “lying flat” posts, and then investigated the associations between mortality salience and the proportions of these specific topics.

## 2 Methods

### 2.1 Data collection

Data on the number of deaths during the COVID-19 pandemic were obtained from the China COVID-19 Knowledge and Data Information System (China Association for Science and Technology, 2023) on a weekly basis.

We collected textual data from Sina Weibo, a popular social media platform in China (Wang et al., 2019). Posts containing the keyword “躺平” (“lying flat”) were retrieved from December 1, 2019, to April 9, 2023, using the Weibo API. A total of 267,073 posts were obtained. Duplicate texts, URLs, and emojis were removed. The remaining 257,410 texts were then tokenized, and stop words were removed^1^. Text preprocessing was conducted using the R package quanteda (Benoit et al., 2018). To exclude the potential impact of socioeconomic factors, such as the unemployment rate, we collected data on the unemployment rates of individuals aged 16–24 from the National Bureau of Statistics (National Bureau of Statistics, 2023).

### 2.2 Data analyses

#### 2.2.1 Relationship between mortality salience and “lying flat”

A stratified regression approach was used to investigate to what extent the number of deaths is associated with the frequency of mentioning “lying flat.” The first stratum included a control variable (i.e., the unemployment rate). We entered the number of deaths as the indicator of mortality salience in the second stratum. This regression model was specifically designed to test Hypothesis 1, where a significant positive coefficient for the number of deaths would provide empirical evidence for the association between mortality salience and the overall frequency of “lying flat” responses.

#### 2.2.2 Topic modeling

While topic modeling has gained widespread acceptance and use in social science research, traditional approaches such as LDA (Blei et al., 2003) may not consistently produce topics that align with the target concepts, resulting in outputs that lack interpretability (Ying et al., 2022). This is because these topic models are fully automatic and do not incorporate input from researchers (Eshima et al., 2023). Consequently, the target concept may not be measurable using these approaches. To avoid this issue and ensure theoretical validity, we utilized a keyword-assisted topic model (Eshima et al., 2023) to uncover the defensive and adaptive “lying flat” topics in the collected posts. One benefit of this approach is that it enables researchers to input keywords related to the target concepts before model fitting. This gives researchers greater control over the topics.

To achieve this, pre-defined keywords were generated for the two target topics, respectively (i.e., the defensive and adaptive “lying flat” topics) based on a multi-staged approach proposed by Eisele et al. (2023). More specifically, we extracted keywords from existing “lying flat” studies, as well as from the authors’ expertise. We further filtered some words from the keywords list and looked for words that appeared within the collected data. Finally, we prepared a list of words occurring frequently within the corpus and scanned for terms that may be useful as markers of the target topics (see the Supplementary material for details). This keyword selection process acted as an initial validation step, ensuring the model’s outputs aligned conceptually with established psychological constructs.

To ensure the reliability and stability of our classification, we systematically evaluated the optimal number of topics. By iteratively fitting the model with topic numbers ranging from 3 to 20, we qualitatively assessed the outputs for semantic coherence, mutual exclusivity and conceptual interpretability across variations (see Karell et al., 2023, for a similar approach). The output topic proportions were used for further analysis. Specifically, we did a correlation analysis with the number of deaths, frequency of posts mentioning “lying flat,” the unemployment rate for individuals aged 16–24, the proportion of the COVID-19 topic^2^, and the proportions of defensive and adaptive topics. Subsequently, we repeated the stratified regressions again but treated the proportion of defensive and adaptive topics as dependent variables, respectively. These subsequent regression models served to validate Hypothesis 2 and Hypothesis 3 by evaluating whether mortality salience is positively associated with the proportions of the defensive and adaptive dimensions of “lying flat” responses.

## 3 Results

### 3.1 Relationship between mortality salience and the frequencies of mentioning “lying flat”

The stratified regression analysis results showed significant differences among the strata. Compared to Model 1, we found a significant incremental *ΔR^2^* of 0.044, *ΔF* (1, 174) = 17.156, *p* < 0.001 in Model 2, in which the number of deaths was added. The coefficients analysis revealed that the frequency of mentioning “lying flat” was significantly associated with the number of deaths (*β* = 0.21, *p* < 0.001), thus providing support for Hypothesis 1. The results of this analysis are presented in Table 1.

**Table 1:** Stratified regression analysis for the frequency of mentioning “lying flat” on the number of deaths caused by COVID-19.

Model	*β*	*SE*	*t*	*F*	*∆R^2^*	*Sig.*
Model 1						
Unemployment rate for individuals aged 16–24	0.69	13.22	13.06^***^	170.66^***^	0.48	0.000^***^
Model 2						
Number of deaths	0.21	0.01	4.14^***^	101.37^***^	0.04	0.000^***^
Unemployment rate for individuals aged 16–24	0.65	12.89	12.66^***^			

***p < 0.001.

### 3.2 Topic modeling

We identified 6 topics (i.e., 3 with the keywords and 3 without) that produced the most coherent, exclusive, and sensible topics. The topic model was finally fitted with the lists of keywords. The model returned the proportion of all topics (aggregated by weeks). Apart from the target topics (i.e., the defensive and adaptive topics) and the third topic related to COVID-19, the remaining three topics were not included in further analyses. The results of the top 10 keywords and representative original texts for the topics are presented in the Supplementary material.

### 3.3 Association among mortality salience, defensive and adaptive “lying flat” topics

**Figure 1:**
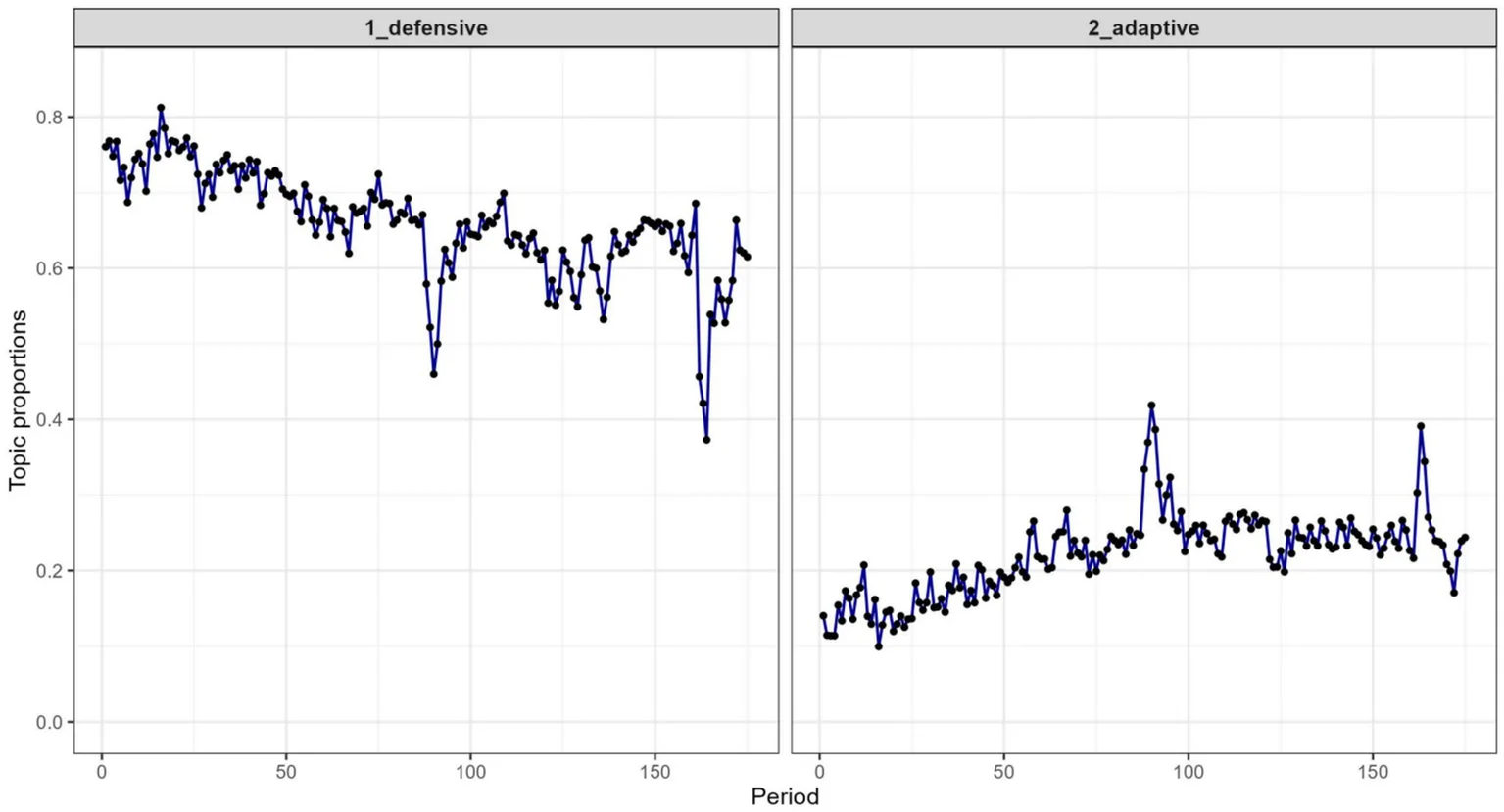
Prevalence of defensive and adaptive topics across the time span of 175 weeks.

This research employed topic modeling to investigate the prevalence of defensive and adaptive topics across a span of 175 weeks, spanning from December 1, 2019, to April 9, 2023. The variations of the two topics are presented in Figure 1.

The correlation analysis indicated that there was no significant correlation between the number of deaths and the proportion of either defensive or adaptive topics. However, both the frequency of mentioning “lying flat” and the proportion of the COVID-19 topic were significantly correlated with the proportion of defensive and adaptive topics (see Table 2).

**Table 2:** Correlation analysis of the number of deaths, the frequency of mentioning “lying flat” and the proportion of the three pre-defined topics.

Model	Number of deaths	Frequency of mentioning “lying flat”	Proportion of COVID-19 topic	Proportion of defensive topic
Frequency of mentioning “lying flat”	0.33^***^			
Proportion of COVID-19 topic	0.21^**^	0.43^**^		
Proportion of defensive topic	−0.11	−0.64^***^	−0.70^**^	
Proportion of adaptive topic	0.01	0.60^***^	0.34^**^	−0.83^**^

**p < 0.01, ***p < 0.001.

Given these intercorrelations, to examine the association between mortality salience and the outcomes while accounting for potential confounding effects, we incorporated the frequency of mentioning “lying flat”and the proportion of COVID-19-related topics as extra control variables. Results indicated that, compared to Model 1, we found a significant incremental *ΔR^2^* of 0.02, *p* = 0.001 in Model 2, in which the number of deaths caused by COVID-19 was added. Results indicated that the proportion of the defensive “lying flat” topic was positively associated with the number of deaths (*β* = 0.15, *p* = 0.001), thus providing support for Hypothesis 2. These results are presented in Table 3.

**Table 3:** Stratified regression analysis for the proportion of the defensive topic on the number of deaths.

Model	*β*	*SE*	*t*	*F*	*∆R^2^*	*Sig.*
Model 1						
Unemployment rate for individuals aged 16–24	0.09	0.00	1.33	102.16^***^	0.62	0.000^***^
Frequency of mentioning “lying flat”	−0.47	0.00	−7.31^***^			
Proportion of COVID-19 related topic	−0.53	0.12	−10.36^***^			
Model 2						
Number of deaths	0.15	0.00	3.28^***^	83.41^***^	0.02	0.001^***^
Unemployment rate for individuals aged 16–24	0.10	0.00	1.65			
Frequency of mentioning “lying flat”	−0.53	0.00	−8.11^***^			
Proportion of COVID-19 related topic	−0.54	0.12	−10.88^***^			

***p < 0.001.

In contrast, when treating the proportion of adaptive “lying flat” topic as the dependent variable, compared to Model 1, we found a significant incremental *ΔR^2^* of 0.05, *p* < 0.001 in Model 2, in which the number of deaths caused by COVID-19 was added. Interestingly, contrary to Hypothesis 3, results indicated that the proportion of the adaptive “lying flat” topic was negatively associated with the number of deaths (*β* = −0.23, *p* < 0.001). These results are presented in Table 4.

**Table 4:** Stratified regression analysis for the proportion of adaptive topic on the number of deaths.

Model	*β*	*SE*	*t*	*F*	*∆R^2^*	*Sig.*
Model 1						
Unemployment rate for individuals aged 16–24	−0.03	0.00	−0.31	35.24^***^	0.37	0.000^***^
Frequency of mentioning “lying flat”	0.57	0.00	6.85^***^			
Proportion of COVID-19 related topic	0.10	0.02	1.55			
Model 2						
Number of deaths	−0.23	0.00	−3.76^***^	31.85^***^	0.05	0.000^***^
Unemployment rate for individuals aged 16–24	−0.05	0.00	−0.66			
Frequency of mentioning “lying flat”	0.66	0.00	7.85^***^			
Proportion of COVID-19 related topic	0.13	0.12	1.94			

***p < 0.001.

## 4 Discussion

While previous research has predominantly focused on the socioeconomic drivers of “lying flat,” the current study provides a psychological account of this phenomenon by examining its existential underpinnings. Our results provide consistent evidence for the primary expectation (Hypothesis 1) that mortality salience, as reflected in COVID-19 death counts, is positively associated with the frequency of “lying flat” responses on social media. More importantly, our fine-grained analysis of specific “lying flat” dimensions yielded mixed support for our predictions. In line with Hypothesis 2, mortality salience was positively associated with the proportion of defensive “lying flat” topics. However, contrary to the initial prediction for the adaptive dimension (Hypothesis 3), higher mortality salience was associated with a decrease, rather than an increase, in the proportion of adaptive topics. These findings suggest that existential threats are associated with the overall movement, particularly with stronger defensive responses in the immediate context of a macro-level crisis.

The positive association between mortality salience and the frequency of mentioning “lying flat” on social media is noteworthy. While previous research highlighted similar withdrawal in specific high-stress populations (e.g., Griffith, 2020; Hu et al., 2020; Sliter et al., 2014), such as frontline nurses (Hu et al., 2020), our findings demonstrate that this psychological response extends to the broader public through digital expression. This suggests that “lying flat” is not merely a reaction to economic stagnation, but a fundamental psychological realignment in the face of overwhelming stress. By vocally rejecting intensive goals, individuals may be engaged in a form of cognitive resource conservation. When the perceived threat to survival increases (e.g., rising death tolls), reducing personal desires and ambition may serve as a vital existential buffer. More specifically, by adopting the idea of “lying flat,” individuals reduce their desires and adopt a less demanding lifestyle, which may provide a sense of security that survival remains attainable without exerting maximum effort.

Noticeably, in our study, we found a positive association between mortality salience and the defensive aspects of “lying flat” but a negative association between mortality salience and adaptive aspects of “lying flat.” One plausible explanation lies in the indirect and mediated nature of the pandemic experience for the general public on social media. Unlike concrete mortality salience, which typically stems from imminent physical threats or near-death experiences (Cozzolino, 2006), the mortality salience triggered by daily death tolls and news reports is inherently abstract. For the majority of users, the pandemic was experienced primarily as informational statistics rather than a physical confrontation with death. According to dual existential systems theory, abstract mortality salience is more likely to elicit defensive responses, whereas concrete mortality salience tends to trigger adaptive responses (Cozzolino, 2006). We therefore speculate that social media primarily serves as an abstract “subtle reminder of death” (Pyszczynski et al., 2004), which may be associated with an increase in the proportion of the defensive “lying flat” topic.

Additionally, it may take different amounts of time to develop adaptive and defensive responses when individuals confront mortality salience. Existing research has indicated that the development of adaptive responses necessitates a longer time frame than that of defensive ones (Sawyer et al., 2010; Cozzolino et al., 2004). This could be attributed to the intricate psychological processes individuals undertake after stress (e.g., Park and Fenster, 2004). It might begin with a defensive and avoiding response, but eventually pave the way for adaptation and personal growth. In the present study, the data collection ended in April 2023, a period when COVID-19-related fatality remained high in China. During this stage, it is plausible that adaptive responses among individuals might not have fully materialized, which may account for the lower proportion of the adaptive topic. This transition seems to be observable in the longitudinal trends: as depicted in Figure 1, there appears to be a linear trend toward an increasing proportion of the adaptive topic and a decline in the defensive topic proportion over time. This pattern aligns with the predictions proposed by the dual existential systems theory, indicating a gradual transition from an initial state of defensive reactions to the adoption of more adaptive strategies as individuals confront and eventually reconcile with their existential concerns.

Notably, expressing “lying flat” does not necessarily mean that the person will actually engage in “lying flat” behavior. Furthermore, social media discourse may involve performative expressions, meaning that participating in the “lying flat” trend online may serve as a collective outlet for frustration rather than a reflection of a genuine, individual-level psychological state. Despite these verbal expressions (i.e., posting “lying flat” topics on social media), individuals may still demonstrate commitment and effort in their work and life. This is supported by data from the Chinese National Bureau of Statistics, which indicates that the working hours of young populations overall have not decreased during COVID-19, but have consistently exceeded 45 h per week (National Bureau of Statistics, 2022). The discrepancy between “lying flat” expression and actual behavior can be attributed to various factors, such as the economic constraints that prevent people from taking time off work. In this sense, future studies are needed to further examine under what conditions individuals may actually perform “lying flat” behavior.

Although this study focuses specifically on the “lying flat” movement on Chinese social media, a similar phenomenon of youth withdrawal has been observed globally, as evidenced by the hikikomori among Japanese youth (Rooksby et al., 2020), the NEET (Not in Education, Employment, or Training) in Western countries (Bynner and Parsons, 2002), and the “Great Resignation” observed among Generation Z in the United States (Jiskrova, 2022). These instances collectively highlight a prevalent disengagement or stagnation among younger generations across different regions (Zhou, 2022). It is therefore essential to examine and expand our findings in the context of cultural differences between Eastern and Western societies. Within the paradigm of Chinese Confucian culture, where the pursuit of career success through effort carries ethical weight, the lack of effort is therefore construed as a moral transgression (Fwu et al., 2014). However, this may not always be the case in other cultures. Hence, cross-cultural comparisons may be crucial in revealing whether the expression of such withdrawal varies among different cultures and whether mortality salience remains the factor triggering this withdrawal phenomenon in different cultural contexts.

This study has several limitations. First, mortality salience was operationalized using COVID-19 death counts as a measure at the aggregate level. Although prior research suggests that such indicators can reflect collective mortality awareness (e.g., Rodriguez et al., 2022), this approach cannot capture individual differences in subjective perception and psychological experience. Therefore, the findings should be interpreted as reflecting macro-level associations rather than individual-level psychological processes. Second, the data were collected exclusively from Weibo, which may limit the generalizability of the findings. While Weibo represents a major platform for public discourse in China, users of this platform may not fully represent the broader population. Future research could incorporate multiple data sources or platforms, such as short-video platforms, to enhance external validity.

## 5 Conclusion

To conclude, in this study, we examined the association between mortality salience and the “lying flat” movement on the Chinese social media platform Weibo. We found that mortality salience was associated with an increased frequency of mentioning “lying flat.” We also discovered that individuals predominantly responded with the defensive “lying flat” topic, and the mortality salience as reflected in COVID-19 was associated with an increased proportion of the defensive “lying flat” topic and a decreased proportion of the adaptive “lying flat” topic. The findings advance our understanding of the psychological underpinnings of “lying flat,” elucidate the complex link between mortality salience and the “lying flat” phenomenon, and highlight the pivotal role of existential concerns in shaping contemporary societal attitudes toward work and well-being.

## Data Availability

The raw data supporting the conclusions of this article will be made available by the authors, without undue reservation.

## Footnotes

1 The stop word list was retrieved from https://github.com/yuanjie-ai/stopwords-zh.

2 We generated a topic specifically related to COVID-19 to ensure that keywords from this topic did not contaminate the two other topics that we analyzed subsequently.
