# Supplementary material for: Deciphering the association between mortality salience and the “lying flat” movement: a social media analysis
Source: Front Psychol. 2026 Aug 7;17:1854408. doi: 10.3389/fpsyg.2026.1854408 (PMC13493589; doi:10.3389/fpsyg.2026.1854408)
Supplement: Supplementary file 1 [file Table_1.DOCX]

Supplementary Material

***The keywords selection***

The selected keywords for the defensive topic are “Slacking (摆烂)”, “Exhausted (好累)”, “Bitterness (苦涩)”, “Anxiety (焦虑)”, “Indulgence (放纵)”, “ Not wanting (不想)”, and “Please sympathize with me (允悲)”; the adaptive topic: “Ordinary (平凡)”, “Reconciliation (和解)”, “Work hard (努力)”, “Life (人生)”, “Choice (选择)”, “Persistence (坚持) ”, “Contentment (知足) ”. We added an additional topic about the Covid-19 pandemic, represented by “Pandemic” (疫情), “COVID-19” (新冠), “virus” (病毒), “zero-Covid” (清零), “infection” (感染) and “Prevention and Control (防控)”.

***Topic modeling results of the pre-defined topics***

| **Labels** | **Top Words** | **Examples** |
| --- | --- | --- |
| Defensive lying flat | 今天 (Today) 不想 (Not wanting) [✓] 感觉 (Feel) 每天 (Every day) 允悲 (Please sympathize with me) [✓] 喜欢 (Like) 工作 (Work) 回家 (Go home) 晚上 (Evening) 苦涩 (Bitterness) [✓] | CN:… 烦心事一大堆，好想躺平什么都不管…  EN:… I have so many worries. I just want to lie flat and ignore everything... |
| Adaptive lying flat | 生活 (Life) 努力 (Work hard) [✓] 工作 (Work) 人生 (Life) [✓] 选择 (Choices) [✓] 事情 (Things) 喜欢 (Like) 孩子 (Children) 需要 (Need) 时间 (Time) | CN:…内卷事实上并不是一个好的情况，他不是说社会中的人都有了上进心和拼搏的干劲，而是说同行之间竞相付出更多的努力，来争取有限资源，导致的个体付出与收益呈现不对等情况的现状。真正的内卷指的是同一层面的纠缠，用爬山来比喻内卷的话，那么以前的社会是多少人付出多少努力爬上多高的山。而内卷的情况则是大家都付出了更多的努力，但是人们就像在走盘山路一样在山脚下或者山的中间不断的循环往复，这样的情况不仅会降低国民的幸福感也会使效率下降，因此很多人将这种情况称之为努力的通货膨胀的体现…  EN: The 'rat race' is not actually a good situation. It does not mean that people in society have developed a drive for progress and hard work, but rather that peers are competing by putting in more effort to fight for limited resources, leading to a disproportionate relationship between individual effort and rewards. The 'rat race' actually refers to entanglement at the same level. If we use climbing a mountain as a metaphor, in the past, the effort people put into determining how high they could climb. However, in the case of the 'rat race,' everyone is putting in more effort, but people are like walking on a mountain road, endlessly looping at the middle of the mountain. This situation not only reduces the happiness of the populace but also decreases efficiency. Therefore, many people describe this phenomenon as a manifestation of the inflation of effort… |
| Pandemic | 疫情 (Epidemic) [✓], 中国 (China) 国家 (Country) 病毒 (Virus) [✓], 美国 (United States) 新冠 (COVID-19) [✓], 防控 (Prevention and control) [✓], 经济 (Economy) 问题 (Issues) 感染 (Infection) [✓] | CN: …国务院联防联控机制11月12日召开新闻发布会，介绍优化调整疫情防控措施，全力抓好当前疫情防控处置工作有关情况。一起来看。二十条优化措施不是放松防控，更不是放开、“躺平”…  EN: On November 12, the State Council Joint Prevention and Control Mechanism held a press conference to introduce the optimization and adjustment of epidemic prevention and control measures, and to provide updates on the current epidemic prevention and control efforts. Let's take a look. The twenty optimization measures are not a relaxation of control, nor are they about opening up or 'lying flat.' |

Note: The keywords marked with a checkmark (✓) are the pre-defined keywords

***Topic modeling results of the remaining topics***

Top words and original texts of remaining topics

| Labels | Top Words | Examples |
| --- | --- | --- |
| Car and vehicle | 凯捷 (Victory) 五菱 (Wuling) 座椅 (Seats) 空间 (Space) 设计 (Design) 二排 (Second row) 伸直 (Stretch out) 配置 (Configuration) 五指 (Five fingers) 体验 (Experience) | CN: …这款家用车空间大颜值高，关键是舒适 对于大多数的中国消费家庭而言，不太在乎那些华而不实的东西，更注重产品的实用性和好品质，譬如说买车，对于有实际消费需求的家用车，更关注车辆的空间性、舒适性和安全性。基于这个准则，如果预算10万左右，购买一辆家用车的话，近期上市的五菱凯捷会是一个不错的选择…  EN: …This family car offers ample space and a stylish appearance, with comfort being the key feature. For most Chinese families, flashy features are not important; they value practicality and high quality. When it comes to buying a car, families with real needs pay more attention to the car's space, comfort, and safety. Based on these criteria, if the budget is around 100,000 RMB, the recently launched Wuling Victory would be a good choice... |
| Misconduct Allegations at a bank | 花椒 (Sichuan pepper) 上班 (Go to work) 支行 (Branch) 光大银行 (Everbright Bank) 行长 (President) 身体 (Body) 分行 (Sub-branch) 医生 (Doctor) 南昌 (Nanchang) 客户 (Customer) | CN:…光大银行南昌分行高新区支行行长助理xx经常旷工逃班居然不撤职 本人于2020年12月30日在光大银行任职期间向光大银行总行及光大银行南昌分行通过邮件方式实名举报了光大银行南昌分行高新区支行行长助理xx经常旷工逃班和工作作风问题。…  EN: The assistant to the president of the High-Tech Zone Branch of Everbright Bank's Nanchang Branch, xx, often skips work without being dismissed. On December 30, 2020, while employed at Everbright Bank, I reported via email to Everbright Bank's headquarters and the Nanchang Branch that xx frequently skipped work and had issues with their work ethic. … |
| Allegations of Judicial Misconduct and Abuse of Power | 新闻 (News) 广西 (Guangxi) 玉州区 (Yuzhou District) 检察 (Prosecution) 玉林市 (Yulin City) 玉林 (Yulin) 严重 (Serious) 刑事 (Criminal) 刑初 (Criminal first instance) 第三 (Third) | CN: … 697号案中依法无罪的举报人，广西玉林市公安局玉州分局敢违法拘捕、玉州区人民检察院敢违法起诉、玉州区人民法院更敢违法审判、判决！…  EN: …In case No. 697, the whistleblower, who was found not guilty according to the law, was illegally arrested by the Yuzhou Branch of the Yulin Public Security Bureau, illegally prosecuted by the Yuzhou District People's Procuratorate, and illegally tried and sentenced by the Yuzhou District People's Court… |
